# Responsiveness of the MOS-HIV and EQ-5D in HIV-infected adults receiving antiretroviral therapies

**DOI:** 10.1186/1477-7525-11-42

**Published:** 2013-03-12

**Authors:** Albert W Wu, Kristin A Hanson, Gale Harding, Seema Haider, Margaret Tawadrous, Alexandra Khachatryan, Chris L Pashos, Kit N Simpson

**Affiliations:** 1 Johns Hopkins Bloomberg School of Public Health, 615 North Wolfe Street, Baltimore, MD, 21205, USA; 2 United BioSource Corporation, 185 Dorval Avenue, Suite 500, Dorval, QC, H9S 5J9, Canada; 3 United BioSource Corporation, 7101 Wisconsin Avenue, Suite 600, Bethesda, MD, 20814, USA; 4 Pfizer Inc, 558 Eastern Point Road, Groton, CT, 06340, USA; 5 Pharmerit, 4350 East West Highway, Suite 430, Bethesda, MD, 20814, USA; 6 United BioSource Corporation, 430 Bedford Street, Suite 300, Lexington Office Park, Lexington, MA, 02420, USA; 7 Medical University of South Carolina, 171 Ashley Avenue, Charleston, SC, 29425, USA

**Keywords:** HIV, Patient-reported outcome (PRO), MOS-HIV, EQ-5D, NNRTI

## Abstract

**Background:**

Selection of an appropriate patient-reported outcome (PRO) instrument for a clinical trial requires knowledge of the instrument’s responsiveness to detecting treatment effects. The purpose of this study was to examine the responsiveness of two health-related quality of life (HRQL) instruments used in clinical trials involving HIV-infected adults: the HIV-targeted Medical Outcomes Study HIV Health Survey (MOS-HIV), and a generic measure, the EuroQol-5D (EQ-5D).

**Methods:**

A systematic review identified clinical trials using the MOS-HIV or EQ-5D to assess outcomes for HIV-infected adults. Data abstracted from each study included study type, treatment regimen(s), PRO results, and effect size (either reported or calculated). Effect size was calculated as the difference between baseline and follow-up mean scores divided by the baseline standard deviation. Magnitude was categorized as small (d=0.20), medium (d=0.50), and large (d=0.80).

**Results:**

Between 2005 and 2010, the MOS-HIV was administered in 12 trials. Significant differences were observed between groups and over time in physical health summary (PHS) and mental health summary (MHS) scores (*P*<0.05) in subjects switching therapy after experiencing Grade-2 adverse events. Effect sizes were medium (0.55 and 0.49 for PHS and MHS, respectively) among treatment-naïve adults beginning therapy (two studies), but negligible among treatment-experienced adults (0.04 and 0.13 for PHS and MHS, respectively; three studies). The EQ-5D was used in five trials between 2001 and 2010. It was responsive to occurrences of adverse events and opportunistic infections, with small-to-medium effect sizes (range 0.30–0.50) in each of its five dimensions.

**Conclusions:**

A systematic review of PRO study results showed both the MOS-HIV and EQ-5D were responsive to changes between groups and/or over time in treatment-naïve HIV-infected patients. These instruments may be used either individually or together in clinical trials to measure changes in HRQL.

## Introduction

The use of highly active antiretroviral therapy (HAART) has improved survival of persons with HIV infection to the extent that HIV-disease is now considered a chronic condition, with treatment goals focused on optimizing health-related quality of life (HRQL) rather than only on improving survival. Therefore, understanding the impact of HAART regimens on HRQL has become increasingly important to patients and their healthcare providers. Furthermore, as regulatory requirements for drug approval have become more stringent [1], authorities are paying close attention to the use of HRQL measures in clinical trials and the subsequent claims that are made based on the trial results.

A comprehensive review of the literature by Clayson et al. (2006) [2] identified and evaluated all HRQL instruments—both generic and HIV-targeted—reported in the HIV/AIDS literature between 1990 and 2005. We conducted an updated and more focused search for HRQL instruments used in clinical trials evaluating non-nucleoside reverse-transcriptase inhibitors (NNRTI)-based regimens from 2005–2010. We then selected one HIV-targeted HRQL instrument, the Medical Outcomes Study HIV Health Survey (MOS-HIV), and one generic HRQL instrument, the EQ-5D, for detailed assessment. Both instruments are widely used in clinical trials and observational studies and are translated into more than 20 languages [3,4]. While the MOS-HIV was the first HIV-targeted instrument developed specifically for use in HIV/AIDS populations, the EQ-5D has also been used patients with advanced HIV disease, typically alongside one or more HIV-specific measures.

Given the growing importance of HRQL in HIV-infected patients while remaining cognizant of the burden associated with administering PRO instruments in clinical trials, it is important to carefully evaluate and select the most sensitive and appropriate HRQL measures for implementation in clinical trials. Therefore, the study was conducted to understand the responsiveness of the MOS-HIV and EQ-5D instruments in clinical trials of HIV-infected adults.

## Methods

### Literature search

A systematic review identified clinical trials administering the MOS-HIV or EQ-5D and evaluating HIV-infected adults from 2005–2010, or earlier when fewer than five studies were identified during that period, as was the case with the EQ-5D. Since only three studies were identified from 2005–2010, the search for EQ-5D studies was expanded to 2001–2010, so that at least five studies could be reviewed. Our search strategy included a combination of Medical Subject Headings (MeSH) terms for HIV [HIV OR HIV infections], instrument names [Euroqol, EQ-5D, MOS-HIV], and clinical trial Publication Types [clinical trial OR clinical trial, phase IV OR clinical trial, phase III OR clinical trial, phase II OR controlled clinical trial OR randomized controlled trial]. We limited our search to articles written in English with abstracts available. In addition to the PubMed search, we conducted a manual search of the bibliographies of the electronically-identified primary studies and review articles.

### Study selection

The inclusion and exclusion criteria for studies to be included in our systematic review were established prior to conducting the literature search. Reviews, editorials, animal studies, and those reporting results of children were excluded from our analysis. All identified articles were initially screened by two authors to exclude duplicates, citations that were clearly irrelevant, and those which did not contain the PRO instruments of interest.

### Data extraction

Data abstracted from each study included study type, treatment regimen(s), PRO results, and effect size (either reported or calculated). Effect size was calculated as the difference between baseline and follow-up mean scores divided by the baseline standard deviation and was interpreted as small (d=0.20), medium (d=0.50), and large (d=0.80) [5]. Statistical significance of results is presented as reported in the original studies; the authors did not calculate or estimate the statistical significance of findings. Where possible, results are aggregated and summarized across studies. Additional results are summarized and presented by study design (randomized controlled trial and non-randomized controlled trials).

### Description of instruments

The MOS-HIV can be administered via survey or interview in approximately 5–10 minutes. The MOS-HIV assesses ten dimensions of HRQL encompassing the following scales: general health perceptions, physical functioning, role functioning, social functioning, pain, energy/fatigue, health distress, mental health, cognitive functioning, and overall quality of life [6]. The scales of the MOS-HIV are scored as summated rating scales on a 0–100 scale where higher scores indicate better health [7].

Combining some of the dimensions, MOS-HIV physical health summary (PHS) and mental health summary (MHS) scores are also generated on a scale of 0–100, with higher scores indicating better health status [8]. The use of summary index scores rather than multiple scale scores simplifies data analysis and the interpretation of findings from clinical trials and aids in comparisons across studies [9]. While all scales contribute to the calculation of the PHS and MHS scores, certain scale scores contribute most strongly. Specifically, the physical function, pain, and role function scale scores contribute most strongly to the PHS score, and the mental health, health distress, quality of life, and cognitive function scales contribute most strongly to the MHS score. The vitality, general health and social function scales contribute to both factors. Summary scores are transformed to t-scores with a mean of 50 and a standard deviation of 10 [7].

The EQ-5D, developed by the EuroQol Group and originally referred to as the Euroqol instrument, is a five-item instrument with one question assessing each of five dimensions: mobility, self-care, usual activities, pain/discomfort, and anxiety/depression. In the version of the EQ-5D used in the studies assessed here, each of the five EQ-5D dimension has three levels, ranging from ‘no problems’ to ‘extreme problems’. Reponses are coded 1, 2, or 3 for each of the dimensions to establish an individual’s health state; there are a total of 243 health states for all possible response combinations. EQ-5D health states may be converted into a summary index by applying a formula that attaches weights to each of the levels in each dimension and deducting the appropriate weights from a score of 1 [10]. Index scores range from 0 to 1 where higher scores indicate better health. The EQ-5D may also include a visual analog scale (VAS) that assesses overall health. VAS scores range from 0 to 100 and higher scores indicate better health.

To improve the instrument’s sensitivity and reduce ceiling effects, the EuroQol Group recently introduced a five level version of the EQ-5D, named the EQ-5D-5L [11]. However, all studies reported in this review utilized the three level (EQ-5D-3L) version of the instrument; hence, all references to the EQ-5D in this review refer to the three level version of the instrument.

## Results

### MOS-HIV

Between 2005 and 2010, the MOS-HIV was administered in 12 clinical trials (nine randomized and three non-randomized prospective controlled trials). Summarized across studies, the MOS-HIV demonstrated the ability to detect change over time in both physical and mental health summary scores among treatment-naïve adults initiating antiretroviral (ARV) therapy (mean effect sizes 0.55 and 0.49, respectively). This was not seen uniformly, however, as effect sizes were negligible in three HIV studies evaluating therapy modifications in treatment-experienced adults (Table 1).

**Table 1:** Summary of MOS-HIV effect sizes overall and in treatment naïve and treatment-experienced HIV-infected patients

**Study**	**Treatment**	**Physical health summary**			**Mental health summary**		
		**Mean change**	**% change**	**Effect size**	**Mean change**	**% change**	**Effect size**
Bucciardini et al. 2007 [13]	ddI/d4T/EFV	4.7	9.4%	−0.43	4.0	8.2%	−0.40
	ddI/d4T/NFV	0.7	1.5%	−0.05	2.8	5.8%	−0.28
	ddI/d4T/EFV/NFV	2.0	4.2%	−0.17	0.0	0.0%	0.00
Stangl et al. 2007^a^[23]	HAART + weekly home visits	15.0*	38.3%	−1.53	14.2*	35.5%	−1.27
**Average for treatment-naïve patients [weighted]**		**2.5**	**0.1**	**−0.55**	**2.3**	**0.1**	**−0.49**
Nuesch et al. 2009^b^[17]	Continuous treatment	−1.3	−2.4%	0.20	0.8	1.5%	0.03
	Scheduled treatment interruptions^c^	−1.2	−2.2%	0.19	0.4	0.8%	−0.15
Huang et al. 2008 [14]	TPV/r (tipranavir / ritonavir)	0.2	0.4%	−0.02	1.4	2.9%	−0.13
	CPI/r (boosted comparitor PI)	−0.3	−0.6%	0.03	1.7	3.6%	−0.17
Powers et al. 2006 [18]	Intermittent group^d^	0.6	1.1%	−0.07	3.2*	5.9%	−0.37
	Continuous group^e^	1.0	1.9%	−0.09	3.2	5.9%	0.00
**Average for treatment-experienced patients [weighted]**		**−0.2**	**0.0**	**0.04**	**1.5**	**0.0**	**−0.13**
**Overall mean [weighted]**		**0.7**	**0.1**	**−0.19**	**1.8**	**0.1**	**−0.27**

Mean change calculated as final score – baseline score. % change calculated as [(final score - baseline score) / baseline score] × 100. Effect size calculated as (final score – baseline score) / standard deviation of baseline score.

*P<0.05; Note: significance of mean change scores not available for all studies; ^a^Prospective non-randomized controlled trial; ^b^Patient population includes treatment-naïve and treatment-experienced patients (analyzed together); ^c^CD4-guided treatment with threshold of 350 cells/μL for interruption/re-initiation of ARV therapy; ^d^7 cycles of 4 weeks off/8 weeks on HAART; ^e^Continue HAART regimen for 22 weeks.

Table 2 presents an overview of MOS-HIV physical and mental health summary scores in the identified studies. Additional study details (e.g., study objective, population characteristics, clinical and PRO results including results of MOS-HIV subscales) are available in the Additional file 1: Appendix. Corresponding with Table 2, results of each of the 12 clinical trials are described in detail below.

**Table 2:** Summary of MOS-HIV physical and mental health summary scores in NNRTI clinical trials

**Citation**	**Treatment/dosing regimen**	**Follow-up period**	**Physical health summary scores**				**Mental health summary scores**			
			**Baseline**	**Follow-up**	**Significance**		**Baseline**	**Follow-up**	**Significance**	
			**Mean (SD)**	**Mean**	**Over time**	**Between groups**	**Mean (SD)**	**Mean**	**Over time**	**Between groups**
**Randomized controlled trials**										
Chang et al. (2007)	Intervention: while receiving individualized acupuncture treatments prescribed by their acupuncturists, listened to tapes with instructions to elicit the relaxation response via earphones, followed by soft music played in the clinic	4 weeks	57.5 (21.1)	61.3	N	N	57.5 (17.0)	62.9	Y	N
		8 weeks		62.7	N	N		62.8	N	N
		12 weeks		65.6	Y	N		68.1	Y	N
	Control: while receiving individualized acupuncture treatments prescribed by their acupuncturists, listened to soft music played in the clinic	4 weeks	62.0 (21.4)	66.7	N	N	64.1 (17.9)	71.0	Y	N
		8 weeks		66.4	N	N		69.5	N	N
		12 weeks		65.7	N	N		70.7	N	N
Bucciardini et al. (2007)	Didanosine + stavudine + efavirenz	1 year	50 (11)	54.7	N	N	49 (10)	53.0	N	N
		2 years		54.9	N	N		50.4	N	N
		3 years		54.9	N	N		49.5	N	N
	Didanosine + stavudine + nelfinavir	1 year	46 (13)	46.7	N	N	48 (10)	50.8	N	N
		2 years		49.2	N	N		51.5	N	N
		3 years		50.9	N	N		53.5	N	N
	Didanosine + stavudine + efavirenz + nelfinavir	1 year	48 (12)	50.0	N	N	50 (9)	50.0	N	N
		2 years		48.1	N	N		49.5	N	N
		3 years		50.0	N	N		53.4	N	N
Huang et al. (2008)	Tipranavir + ritanovir	48 weeks	48.0 (11.3)	48.2	NM	N	47.8 (10.5)	49.2	NM	N
	Boosted comparitor protease inhibitor	48 weeks	47.1 (10.9)	46.8	NM	N	46.9 (10.3)	48.6	NM	N
Lafaurie et al. (2008)	Maintenance of a stable protease inhibitor-containing regimen	48 weeks	NR	−1.04	NM	N	NR	0.00	NM	N
	Switch to efavirenz + didanosine + emtricitabine once-daily	48 weeks	NR	−1.76	NM	N	NR	1.01	NM	N
Sprinz et al. (2006)	Immediate substitution with lopinavir/ritonavir 400/200 mg twice daily	4 weeks	49.44	52.06	Y	Y	46.7	50.79	Y	Y
		8 weeks		52.38	Y	Y		51.34	Y	Y
	Deferred (week 4) substitution with lopinavir/ritonavir 400/100 mg twice daily	4 weeks	50.32	49.71	N	Y	48.25	48.03	N	Y
		8 weeks		51.12	N	Y		50.02	Y	Y
Nuesch et al. (2009)	Continuous treatment	24 weeks	53.8 (6.6)	52.7	N	N	51.7 (7.6)	52.7	Y	Y
		48 weeks		52.5	N	N		52.5	Y	Y
	CD4-guided scheduled treatment interruption (CD4 threshold of 350 cells/μL for interruption/re-initiation of ARV therapy)	24 weeks	54.4 (6.3)	53.2	N	N	49.1 (8.3)	48.1	Y	Y
		48 weeks		53.2	N	N		49.5	Y	Y
Powers et al. (2006)	Intermittent treatment (7 cycles of 4 weeks off/8 weeks on HAART)	4 weeks	54.2 (8.3)	54.2	N	Y	54.0 (8.8)	53.56	N	Y
		12 weeks		55.55	N	Y		57.45	Y	Y
		40 weeks		54.76	N	Y		57.22	Y	Y
	Continuous treatment (continue regimen for 22 weeks)	4 weeks	53.0 (11.5)	53.27	N	Y	53.8 (15.7)	53.36	N	Y
		12 weeks		52.97	N	Y		57.25	N	Y
		40 weeks		54.00	N	Y		57.02	N	Y
Wu et al. (2006)	Disease Management Assistance System + education	6 months	45.7 (11.0)	44.2	NR	Y	49.2 (10.3)*	49.5	NR	N
	Education only	6 months	41.2 (12.7)	47.0	NR	Y	40.7 (12.4)*	44.9	NR	N
**Non-randomized controlled trials**										
Shalit et al. (2007)	Enfuvirtide + ARTs	12 weeks	NR	2.21	Y	N/A	NR	2.91	Y	N/A
Levine et al. (2008)	Epoetin alfa + iron supplementation (in addition to current ART regimen)	MPD1	38.5 (12.0)	43.0	N	N/A	42.4 (11.2)	47.8	N	N/A
		24 weeks		43.5	Y	N/A		47.5	Y	N/A
Stangl et al. (2007)	HAART and weekly home visits by study staff that re-supplied HAART and other drugs, conducted a pill count, and assessed participants’ health	3 months	39.2 (9.8)	50.6	Y	N/A	40.0 (11.2)	50.2	Y	N/A
		6 months		53.0	Y	N/A		53.0	Y	N/A
		9 months		53.6	Y	N/A		54.2	Y	N/A
		12 months		54.2	Y	N/A		54.2	Y	N/A

*significant difference in baseline scores (*P*=0.01).

#### Randomized controlled trials

A study by Chang et al. (2007) [12] evaluated the effect of adding the relaxation response to usual acupuncture treatment in HIV-infected adults. From baseline to 12-week follow-up, the mean MHS score increased 10.6 points (*P*<0.001) and the PHS score increased 8.1 points (*P*<0.01) in the intervention group (*P*<0.01); no significant differences were observed in the control group. In addition, there was a clinically significant seven-point difference in the energy subscale of the MHS.

A study by Bucciardini et al. (2007) [13] compared the initiation of didanosine (ddI)/stavudine (d4T)/efavirenz (EFV) versus ddI/d4T/nelfinavir (NFV) versus ddI/d4T/EFV/NFV in treatment-naïve HIV-infected adults over three years. The trial found both no significant differences over time and no significant differences between treatment groups in MOS-HIV scores.

Three studies evaluated treatment regimens containing protease inhibitors (PIs). Huang et al. (2008) [14] compared tipranavir/ritonavir (TPV/r) versus a comparator protease inhibitor similarly boosted with ritonavir (CPI/r). Of all MOS-HIV domains measured, only one significant difference between groups was observed; compared to CPI/r, TPV/r was associated with a small but significant improvement in pain scores (+4.8 points; *P*<0.05). A study by Lafaurie et al. (2008) [15] evaluated HIV-infected adults receiving a PI-containing ARV regimen and compared maintenance of the current PI-containing regimen versus switch to a once-daily NNRTI-based regimen of EFV/ddI/emtricitabine (FTC). Although a medium effect was observed in the PHS score in the switch arm (mean change −1.76, effect size 0.53), the difference in change from baseline to 48 weeks in both PHS and MHS scores was not statistically significant between treatment groups (*P*=0.57 and *P*=0.42, respectively). Sprinz et al. (2006) [16] evaluated the effect of substituting lopinavir/ritonavir (LPV/r) for the PI component of a HAART regimen in patients experiencing treatment-related Grade-2 side effects. Treatment arms in this study were immediate substitution (IS) versus deferred (week 4) substitution (DS). At week 4, patients in the IS group had improvement of 2.6 points in the PHS score (from a baseline of 49.4; *P*<0.001) and a 4.1 point improvement in the MHS score (from a baseline of 46.7; *P*<0.001). Furthermore, at week 4, the effect size for the between-group difference in PHS and MHS scores were 0.45 and 0.56, respectively, suggesting moderate clinically meaningful improvement in the IS group compared to the DS group. The difference between the groups was statistically significant (*P*<0.001 for both PHS and MHS scores).

Two randomized controlled trials (RCTs) evaluated continuous versus intermittent ARV therapy. A study by Nuesch et al. (2009) [17] compared continuous ARV treatment with scheduled treatment interruption (STI) and showed no significant change in PHS over time or between groups. MHS scores were significantly higher in the continuous treatment group than the STI group at baseline, 24 weeks, and 48 weeks, but not at the final visit. Furthermore, MHS scores significantly improved over time in both groups (*P*=0.001), but the improvement was not significantly different between groups (*P*=0.17). In a RCT by Powers et al. (2006) [18], patients receiving intermittent ARV therapy had significantly higher PHS and MHS scores at baseline and during each follow-up point compared to patients receiving continuous ARV therapy. Compared to baseline, a significant improvement was observed in MHS score at weeks 12 and 40 in patients receiving intermittent ARV therapy; no other significant changes over time were observed.

The final two RCTs did not distinguish between ARV treatment regimens in reporting HRQL results. In a secondary analysis of Options in Management with Antiretrovirals (OPTIMA) data, Anis et al. (2009) [19] evaluated several treatment regimens in multidrug resistant HIV-infected adults and reported MOS-HIV scores for all treatment regimens collectively at baseline and at four follow-up time points, stratifying patients by presence or absence of AIDS-defining events (ADEs), serious adverse events (SAEs), and improvement in clinical measures—CD4 count and viral load. Although significance of MOS-HIV score changes over time was not reported, PHS and MHS scores were significantly lower for those that experienced SAEs or ADEs compared to those who did not at most time points. Similarly, PHS and MHS scores were significantly higher among those with improvement in CD4 count compared to those with no improvement (*P*≤0.01) [see Additional file 1: Appendix]. A study by Wu et al. (2006) [20] compared a Disease Management Assistance System (DMAS) with education versus education only in HIV-infected adults. Although there were no significant differences between the groups at follow-up on all ten MOS-HIV domains, there were significant differences at baseline on five of the scales. The study concluded that the differences between groups generally reflected a combination of improvement in the standard education arm and some deterioration in the DMAS arm over time.

#### Non-randomized controlled trials

All three prospective non-RCTs evaluated a single treatment arm. A study by Shalit et al. (2007) [21] evaluated the addition of enfuvirtide (ENF, T-20) to ARV therapy in treatment-experienced, enfuvirtide-naïve, HIV-infected adults. Significant improvements were observed in the least squares mean (LSM) change from baseline to week 12 scores among most MOS-HIV domains; social function and role function were the only domains in which significant improvements did not occur. A trial by Levine et al. (2008) [22] evaluated the efficacy and safety of the administration of erythropoietin alfa in maintaining HRQL and hemoglobin levels in anemic HIV-infected patients. In this study, all domains of the MOS-HIV improved significantly from baseline to day one of the maintenance phase (all *P*<0.001, except role function *P*<0.05). Improvements in HRQL scores were associated with improvements in hemoglobin levels. Finally, a study by Stangl et al. (2007) [23] evaluated the initiation of HAART and weekly home visits in treatment-naïve HIV-infected adults in rural Uganda. At each follow-up visit, PHS and MHS scores were significantly higher than baseline scores (*P*<0.001) and HRQL improvement closely tracked improvements in CD4 cell count.

### EQ-5D

The EQ-5D was administered in five HIV trials (three RCTs and two prospective observational studies) between 2001 and 2010. Overall, the EQ-5D was responsive to occurrences of adverse events and opportunistic infections, with small-to-medium effect sizes (range 0.3–0.5) in each of its five dimensions. In addition, the EQ-5D demonstrated medium effect sizes in all dimensions in a prospective enfuvirtide study (Table 3). Only one study measured and reported the change in EQ-5D scores over time. A summary of key findings is presented below, group by study design; additional details of each of the 5 trials are available in the Additional file 1: Appendix.

**Table 3:** Effect sizes observed in EQ-5D items in a prospective study of enfuvirtide

**EQ-5D dimension**	**Mean change**	**% change**	**Effect size**
Mobility	−0.4	−26.67%	−0.4
Self-care	−0.2	−16.67%	−0.3
Usual activities	−0.4	−23.53%	−0.5
Pain/discomfort	−0.3	−17.65%	−0.3
Anxiety/depression	−0.5	−26.32%	−0.4

Mean change calculated as final score – baseline score. % change calculated as [(final score - baseline score) / baseline score] x 100. Effect size calculated as (final score – baseline score) / standard deviation of baseline score.

#### Randomized controlled trials

One clinical trial used the EQ-5D to assess quality of life in a study aimed to determine whether side effects of PI-containing ARV therapy, such as lipodystrophy, dyslipidemia, and insulin resistance, are reversible with continued HIV suppression following PI substitution [24]. Eighty-one treatment-experienced patients were randomized to either continue their current PI + nucleoside-based therapy (control patients) or switch the PI-component of their regimen to abacavir/nevirapine/adefovir (plus hydoxyurea at week 4). In this study, both patient-assessed and physician-assessed EQ-5D scores were reported. The change in patient-assessed EQ-5D scores from baseline to 24-week follow-up (−6 in control group versus +8 in switch group) was not statistically significant (*P*=0.074), while the change in physician-assessed EQ-5D scores (−7 in control group versus +8 in switch group) was statistically significant (*P*=0.016).

In a secondary analysis of OPTIMA data, Anis et al. (2009) [19] evaluated several treatment regimens in multidrug-resistant HIV-infected adults and reported EQ-5D scores for all treatment regimens collectively at baseline and at four follow-up time points, stratifying patients by presence or absence of ADEs, SAEs, and improvement in clinical measures—CD4 count and viral load. Similar to the findings of the MOS-HIV, EQ-5D scores were significantly lower for those that experienced SAEs or ADEs compared to those who did not at most time points (all with the exception of ADE at time point one *P*≤0.01). Also similar to the MOS-HIV results, EQ-5D scores were significantly higher among patients with improvement in CD4 count compared to those with no improvement (*P*≤0.05).

**Figure 1:**
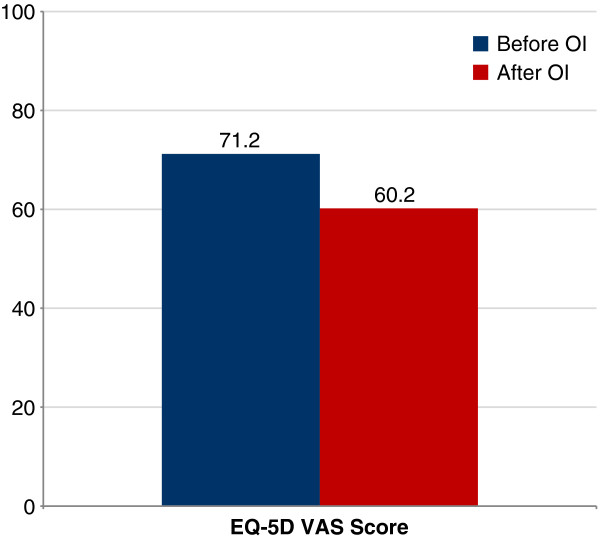
Sensitivity of EQ-5D VAS to detect change in HRQL with experience of an opportunistic infection.

A study by Wu et al. (2002) [4] compared valacyclovir and acyclovir as prophylactic regimens for cytomegalovirus, stratifying EQ-5D results by presence or absence of adverse events, without regard to treatment allocation in the study. At baseline, no patients had the lowest possible EQ-5D score, while 28.2% scored the highest attainable score of 1.0 and 75% of patients scored between 0.72 and 1. Effect size was moderate (0.40) for the EQ-5D Index among patients experiencing an adverse event (Figure 1); effect sizes for all other dimensions were “small” and insignificant (0.05–0.20). In a subgroup of patients experiencing an opportunistic infection (OI), the EQ-5D index score demonstrated no change (0.60 before and after OI diagnosis), whereas the EQ-5D VAS demonstrated a significant decrease from 71.2 to 60.2 after OI diagnosis (*P*<0.01).

#### Prospective observational studies

A small study (n=16) by Bucciardini et al. (2008) [25] evaluated the addition of enfuvirtide to a selected optimized background ARV regimen in HIV-infected adults. On average, the EQ-5D profile of the enrolled subjects improved over time during the six-month follow-up (*P* value not reported). Effect size was moderate (0.50) for the Usual Activities domain at three and six months; effect sizes for all other dimensions were “small” (0.20–0.40) at both three and six months. A similar but larger study (n=102) evaluating the addition of enfuvirtide to a selected optimized background ARV regimen was conducted in South Africa [26]. In this study, mean EQ-5D scores did not change significantly during the 18-month follow-up and effect sizes (0.03–0.05) were negligible.

## Discussion

Over a recent five-year period, the MOS-HIV Health Survey has been one of the most widely used PRO instruments in treatment trials for people with HIV disease. The EQ-5D has also been used, though not as frequently. However, given recent regulatory guidance, we expect these PRO instruments, and others, will be used more in future clinical trials.

It is important to note that it is sometimes difficult to demonstrate PRO responsiveness in the setting of a clinical trial, since there are not always actual differences between groups to be detected. Many recent HAART studies in ARV-naïve patients are designed as equivalence studies; thus, the effect of HIV disease in the study arms may be expected to be quite similar. In addition, if the newer HAART regimens used in these patient groups have similar side effect profiles, except for SAEs that lead to study discontinuation, measurable differences in HRQL would not be expected. Several of the studies that we reviewed used highly effective treatments with similar side effect profiles in both arms. In these cases, it appears that the observed lack of change in PRO scores in the specific studies may be due more to the treatments used in the studies than to the sensitivity of the PRO instruments. However, overall examination of published trial results indicates that both of these PRO measures are responsive to changes in clinical condition in the intended patient population.

Overall, we observed that the MOS-HIV was responsive to changes in HIV-infected patients initiating ARV therapy for the first time. Although we did not find similar responsiveness among treatment-experienced patients, it is important to note that only three studies were reviewed and in each of them only minor ARV therapy modifications were made. Two studies evaluated the clinical and patient-reported effects of treatment interruption [17,18] and the third study compared two boosted PI regimens, which are expected to have similar side effects [14]. Therefore, we are unable to conclude from our review whether or not the MOS-HIV is sensitive to more substantial ARV therapy modifications in treatment-experienced patients, such as switching from an NNRTI-based regimen to a PI-based regimen.

Although a smaller literature base is available for the EQ-5D, this instrument has demonstrated responsiveness to ARV therapy changes [25], occurrences of ADEs [19], AEs [4,19], and OIs [4]. Studies have not been conducted that showed differences in change scores between treatment groups. Advantages of the EQ-5D include its low administrative burden (five items with an optional VAS) and its ability to generate indirect health utility values for use in economic models.

## Conclusions

Our systematic literature review suggests that both the MOS-HIV and EQ-5D instruments are responsive to clinical changes in HIV-infected patients. These two instruments may complement each other and researchers should consider using them together in clinical trials to obtain HIV-specific HRQL and utility measures without excessive respondent burden.

## Abbreviations

ART: Antiretroviral therapy; ddI: Didanosine; d4T: Stavudine; EFV: Efavirenz; HAART: Highly active antiretroviral therapy; HAART: Highly active antiretroviral therapy; MPD1: Maintenance phase day 1, defined as first day patient achieves hemoglobin level ≥13 g/dL; NFV: Nelfinavir; PI: protease inhibitor; MHS: Mental health summary; N/A: Not applicable; NM: Not measured; NR: Not reported; PHS: Physical health summary.

## Competing interests

KAH, GH, and CLP are employees of United BioSource Corporation, which received funding for this research from Pfizer. SH and MT are employees of and have equity ownership in Pfizer. AK was an employee of Pfizer at the time the study was conducted. KNS and AWW received funding for this research from Pfizer.

## Authors’ contributions

KAH and GH participated in the study conception and design, acquisition of data, data analysis and interpretation, and manuscript writing. KNS, SH, MT, CLP, and AWW participated in the study conception and design, data interpretation, and manuscript writing. AK participated in the data interpretation and manuscript writing. All authors read and approved the final manuscript.

## Supplementary Material

Additional file 1 **PRO Responsiveness Manuscript_Appendix Table A and B.doc.** Data extraction tables for MOS-HIV and EQ-5D. Click here for file

## References

[B1] Food and Drug Administration Guidance for industry on patient-reported outcome measures: use in medical product development to support labeling claims Fed Regist 2009 74 65132 65133

[B2] Clayson DJ Wild DJ Quarterman P Duprat-Lomon I Kubin M Coons SJ A comparative review of health-related quality-of-life measures for use in HIV/AIDS clinical trials Pharmacoeconomics 2006 24 751 765 10.2165/00019053-200624080-00003 16898846

[B3] Patient-Reported Outcome and Quality of Life Instruments Database (PROQOLID) http://www.proqolid.org/10.1186/1477-7525-3-12 PMC555954 15755325

[B4] Wu AW Jacobson KL Frick KD Clark R Revicki DA Freedberg KA Scott-Lennox J Feinberg J Validity and responsiveness of the euroqol as a measure of health-related quality of life in people enrolled in an AIDS clinical trial Qual Life Res 2002 11 273 282 10.1023/A:1015240103565 12074264

[B5] Cohen J A power primer Psychol Bull 1992 112 155 159 19565683 10.1037//0033-2909.112.1.155

[B6] Wu AW Hays RD Kelly S Malitz F Bozzette SA Applications of the Medical Outcomes Study health-related quality of life measures in HIV/AIDS Qual Life Res 1997 6 531 554 10.1023/A:1018460132567 9330553

[B7] Wu AW MOS-HIV Health Survey Users Manual 1999 Baltimore, Maryland: Johns Hopkins University

[B8] Wu AW Revicki DA Jacobson D Malitz FE Evidence for reliability, validity and usefulness of the Medical Outcomes Study HIV Health Survey (MOS-HIV) Qual Life Res 1997 6 481 493 10.1023/A:1018451930750 9330549

[B9] Revicki DA Sorensen S Wu AW Reliability and validity of physical and mental health summary scores from the Medical Outcomes Study HIV Health Survey Med Care 1998 36 126 137 10.1097/00005650-199802000-00003 9475468

[B10] Rabin R Oemar M Oppe M EQ-5D-3L User Guide: Basic information on how to use the EQ-5D-3L instrument. Version 4.0 2011 Rotterdam, The Netherlands: EuroQol Group

[B11] Herdman M Gudex C Lloyd A Janssen M Kind P Parkin D Bonsel G Badia X Development and preliminary testing of the new five-level version of EQ-5D (EQ-5D-5L) Qual Life Res 2011 20 1727 1736 10.1007/s11136-011-9903-x 21479777 PMC3220807

[B12] Chang BH Boehmer U Zhao Y Sommers E The combined effect of relaxation response and acupuncture on quality of life in patients with HIV: a pilot study J Altern Complement Med 2007 13 807 815 10.1089/acm.2007.7024 17983336 PMC2917624

[B13] Bucciardini R Fragola V Massella M Polizzi C Mirra M Goodall R Carey D Hudson F Zajdenverg R Floridia M Initio Trial International Coordinating C Health-related quality of life outcomes in HIV-infected patients starting different combination regimens in a randomized multinational trial: the INITIO-QoL substudy AIDS Res Hum Retroviruses 2007 23 1215 1222 10.1089/aid.2007.0067 17961107

[B14] Huang IC Wu AW Finnern HW Thijs H Gathe JC Fairclough DL Health-related quality of life and tolerability in treatment-experienced HIV-1-infected patients on tipranavir versus comparator regimens Antivir Ther 2008 13 15 25 18389895 10.1177/135965350801300102

[B15] Lafaurie M Collin F Bentata M Garre M Leport C Levy Y Goujard C Chene G Molina JM Group AS Switch from zidovudine- to non-zidovudine-containing regimens is associated with modest haematological improvement and no obvious clinical benefit: a substudy of the ANRS 099 ALIZE trial J Antimicrob Chemother 2008 62 1122 1129 10.1093/jac/dkn309 18662943

[B16] Sprinz E Neto AJ Bargman E Green SL Luo MP Sylte JR McMillan FI King KR Rode RA Brun SC Substitution with lopinavir/ritonavir improves patient-reported outcomes including quality of life in patients who were intolerant to their antiretroviral therapy HIV Clin Trials 2006 7 291 308 10.1310/hct0706-291 17197377

[B17] Nuesch R Gayet-Ageron A Chetchotisakd P Prasithsirikul W Kiertiburanakul S Munsakul W Raksakulkarn P Tansuphasawasdikul S Chautrakarn S Ruxrungtham K The impact of combination antiretroviral therapy and its interruption on anxiety, stress, depression and quality of life in Thai patients Open AIDS J 2009 3 38 45 10.2174/1874613600903010038 19812705 PMC2757643

[B18] Powers AE Marden SF McConnell R Leidy NK Campbell CM Soeken KL Barker C Davey RT Dybul MR Effect of long-cycle structured intermittent versus continuous HAART on quality of life in patients with chronic HIV infection AIDS 2006 20 837 845 10.1097/01.aids.0000218547.39339.13 16549967

[B19] Anis AH Nosyk B Sun H Guh DP Bansback N Li X Barnett PG Joyce V Swanson KM Kyriakides TC Quality of life of patients with advanced HIV/AIDS: measuring the impact of both AIDS-defining events and non-AIDS serious adverse events J Acquir Immune Defic Syndr 2009 51 631 639 10.1097/QAI.0b013e3181a4f00d 19430303

[B20] Wu AW Snyder CF Huang IC Skolasky R McGruder HF Celano SA Selnes OA Andrade AS A randomized trial of the impact of a programmable medication reminder device on quality of life in patients with AIDS AIDS Patient Care STDS 2006 20 773 781 10.1089/apc.2006.20.773 17134351

[B21] Shalit P True A Thommes JA Investigators Q Quality of life and tolerability after administration of enfuvirtide with a thin-walled needle: QUALITE Study HIV Clin Trials 2007 8 24 35 10.1310/hct0801-24 17434846

[B22] Levine AM Salvato P Leitz GJ Champs 2 Study G Efficacy of epoetin alfa administered every 2 weeks to maintain hemoglobin and quality of life in anemic HIV-infected patients AIDS Res Hum Retroviruses 2008 24 131 139 10.1089/aid.2006.0200 18284320

[B23] Stangl AL Wamai N Mermin J Awor AC Bunnell RE Trends and predictors of quality of life among HIV-infected adults taking highly active antiretroviral therapy in rural Uganda AIDS Care 2007 19 626 636 10.1080/09540120701203915 17505923

[B24] Carr A Hudson J Chuah J Mallal S Law M Hoy J Doong N French M Smith D Cooper DA Group PS HIV protease inhibitor substitution in patients with lipodystrophy: a randomized, controlled, open-label, multicentre study AIDS 2001 15 1811 1822 10.1097/00002030-200109280-00010 11579243

[B25] Bucciardini R Massella M Corpolongo A Narciso P Fragola V Mirra M Donnini S Vigano O Costarelli S Tozzi V T20QoL: an observational multicenter cohort study to evaluate the quality of life in HIV-patients treated with enfuvirtide (ENF, T-20) in combination with an optimized background therapy Biologics 2008 2 577 581 19707388 10.2147/btt.s3187 PMC2721382

[B26] Bhargava A Booysen Fle R Healthcare infrastructure and emotional support are predictors of CD4 cell counts and quality of life indices of patients on antiretroviral treatment in Free State Province, South Africa AIDS Care 2010 22 1 9 20390475 10.1080/09540120903012585

