# Supplementary material for: Responsiveness of the MOS-HIV and EQ-5D in HIV-infected adults receiving antiretroviral therapies
Source: Health Qual Life Outcomes. 2013 Mar 12;11:42. doi: 10.1186/1477-7525-11-42 (PMC3602001; doi:10.1186/1477-7525-11-42)
Supplement: Additional file 1 — PRO Responsiveness Manuscript_Appendix Table A and B.doc. Data extraction tables for MOS-HIV and EQ-5D. [file 1477-7525-11-42-S1.doc]

**Appendix**

Table A. Data extraction tables for MOS-HIV

| **Citation** | **Study design and objective** | **Study population** | **PRO assessment times and other instruments** | **Treatment/dosing regimen** | **Domain** | **Baseline score** mean (SD) | | **Follow-up score (**time, mean) | **Effect size** | **Significant difference** | | | **Clinical/PRO results** |
| --- | --- | --- | --- | --- | --- | --- | --- | --- | --- | --- | --- | --- | --- |
|  |  |  |  |  |  |  |  |  |  | **Over time** | **Between groups** | |  |
| **RANDOMIZED CONTROLLED TRIALS** | | | | | | | | | | | | | |
| Chang et al. (2007) | • Prospective RCT  • To examine the effects of adding the relaxation response (RR) to usual acupuncture treatment on improving the QoL of HIV/AIDS patients | • N=119 (58 in intervention arm, 61 in control arm) • HIV-infected adults who had at least 1 of the highly prevalent HIV-related symptoms and who were receiving acupuncture treatment | • Baseline  • 4 weeks • 8 weeks  • 12 weeks • Other Instruments: FAHI FACIT-Sp (Functional Assessment of Chronic Illness Therapy--Spiritual Well-being) | Intervention: while receiving individualized acupuncture treatments prescribed by their acupuncturists, listened to tapes with instructions to elicit the relaxation response via earphones, followed by soft music played in the clinic | PHS | 57.5 (21.1) | | 4, 61.3 | -0.18 | No | No | | • At the 12-week follow-up, significant improvements were observed in PHS and MHS in the intervention group (*P*=0.003 and *P*=0.0003, respectively); no significant improvements were observed in the control group.  • There were no statistically significant differences in score increases between the groups over time; however, a marginal significant difference was observed in PHS (*P=* 0.07).  • In this study, an increase of 3.4 points was considered clinically significant because it corresponded to improvement over 4 months in performance status, as measured by the Karnofsky Performance Index, in patients with HIV.  • From baseline to 12-week follow-up, the mean MHS increased 10.6 points in the intervention group; no clinically significant differences were observed in the control group. • A 7-point difference in the energy subscale--one component of MHS--was considered clinically significant as it distinguished between asymptomatic and symptomatic patients with HIV. |
|  |  |  |  |  |  |  |  | 8, 62.7 | -0.25 | No | No | |  |
|  |  |  |  |  |  |  |  | 12, 65.6 | -0.38 | Yes | No (*P*=0.07) | |  |
|  |  |  |  |  | MHS | 57.5 (17.0) | | 4, 62.9 | -0.32 | Yes | No | |  |
|  |  |  |  |  |  |  |  | 8, 62.8 | -0.31 | No | No | |  |
|  |  |  |  |  |  |  |  | 12, 68.1 | -0.62 | Yes | No | |  |
|  |  |  |  | Control: while receiving individualized acupuncture treatments prescribed by their acupuncturists, listened to soft music played in the clinic | PHS | 62.0 (21.4) | | 4, 66.7 | -0.22 | No | No | |  |
|  |  |  |  |  |  |  |  | 8, 66.4 | -0.21 | No | No | |  |
|  |  |  |  |  |  |  |  | 12, 65.7 | -0.17 | No | No (*P*=0.07) | |  |
|  |  |  |  |  | MHS | 64.1 (17.9) | | 4, 71.0 | -0.39 | Yes | No | |  |
|  |  |  |  |  |  |  |  | 8, 69.5 | -0.30 | No | No | |  |
|  |  |  |  |  |  |  |  | 12, 70.7 | -0.37 | No | No | |  |
| Bucciardini et al. (2007) | • Prospective RCT (secondary analysis of INITIO-QoL data)  • To detect differences in patient’s HRQL among the three study treatment groups in the INITIO trial, a large, multinational trial comparing different treatment strategies for starting and continuing HIV treatment in ARV naive patients. In addition, this study aimed to provide, by patient-reported outcomes, potentially relevant additional information alongside clinical data, for making decisions on medical treatment. | • N=139 (44 in ddI/d4T/EFV arm, 47 in ddI/d4T/NFV arm, 48 in ddI/d4T/EFV/NFV arm)  • Treatment-naïve HIV-1-infected adults | • Baseline  • Year 1 • Year 2 • Year 3  • Other Instruments: None | ddI/d4T/EFV | Physical Health (PHS) | 50 (11) | | 1, 54.7 | -0.43 | No | No | | • During follow-up, an increase of PHS score was observed in all treatment arms; results of the general linear mixed model showed no statistically significant differences over time in PHS scores among the treatment arms.  • The MHS score remained substantially unchanged with the four-drug combination and showed with both NNRTI- and PI-based three-drug regimens a marked trend toward improvement, which became statistically significant when a multiple imputation method was used to adjust for missing data.  • Overall, starting all the combination regimens compared in the INITIO study was associated with a maintained or slightly improved HRQL status, consistently with the positive immunological and virological changes observed in the main study.  • The observed differences in the MHS indicate a possible HRQL benefit associated to the use of three-drug, two-class regimens and no additional benefit for the use of four-drug, three-class regimens, confirming that three-drug, two-class regimens that include two NRTIs plus either an NNRTI or a PI should be preferred as initial treatment of HIV infection. |
|  |  |  |  |  |  |  |  | 2, 54.9 | -0.45 | No | No | |  |
|  |  |  |  |  |  |  |  | 3, 54.9 | -0.45 | No | No | |  |
|  |  |  |  |  | Mental Health (MHS) | 49 (10) | | 1, 53.0 | -0.40 | No | No | |  |
|  |  |  |  |  |  |  |  | 2, 50.4 | -0.54 | No | No | |  |
|  |  |  |  |  |  |  |  | 3, 49.5 | -0.05 | No | No | |  |
|  |  |  |  |  | GPH | 42 (27) | | Not reported |  |  |  | |  |
|  |  |  |  |  | Pain | 80 (25) | |  |  |  |  |  |  |
|  |  |  |  |  | PF | 79 (24) | |  |  |  |  |  |  |
|  |  |  |  |  | RF | 78 (37) | |  |  |  |  |  |  |
|  |  |  |  |  | SF | 72 (31) | |  |  |  |  |  |  |
|  |  |  |  |  | MH | 65 (20) | |  |  |  |  |  |  |
|  |  |  |  |  | EF | 56 (24) | |  |  |  |  |  |  |
|  |  |  |  |  | HD | 67 (26) | |  |  |  |  |  |  |
|  |  |  |  |  | CF | 82 (20) | |  |  |  |  |  |  |
|  |  |  |  |  | QOL | 60 (26) | |  |  |  |  |  |  |
|  |  |  |  |  | TH | 62 (27) | |  |  |  |  |  |  |
|  |  |  |  | ddI/d4T/NFV | Physical Health (PHS) | 46 (13) | | 1, 46.7 | -0.05 | No | No | |  |
|  |  |  |  |  |  |  |  | 2, 49.2 | -0.25 | No | No | |  |
|  |  |  |  |  |  |  |  | 3, 50.9 | -0.38 | No | No | |  |
|  |  |  |  |  | Mental Health (MHS) | 48 (10) | | 1, 50.8 | -0.28 | No | No | |  |
|  |  |  |  |  |  |  |  | 2, 51.5 | -0.35 | No | No | |  |
|  |  |  |  |  |  |  |  | 3, 53.5 | -0.55 | No | No | |  |
|  |  |  |  |  | GPH | 38 (28) | | Not reported |  |  |  | |  |
|  |  |  |  |  | Pain | 76 (24) | |  |  |  |  |  |  |
|  |  |  |  |  | PF | 70 (30) | |  |  |  |  |  |  |
|  |  |  |  |  | RF | 67 (45) | |  |  |  |  |  |  |
|  |  |  |  |  | SF | 75 (31) | |  |  |  |  |  |  |
|  |  |  |  |  | MH | 63 (23) | |  |  |  |  |  |  |
|  |  |  |  |  | EF | 55 (22) | |  |  |  |  |  |  |
|  |  |  |  |  | HD | 71 (26) | |  |  |  |  |  |  |
|  |  |  |  |  | CF | 78 (23) | |  |  |  |  |  |  |
|  |  |  |  |  | QOL | 56 (28) | |  |  |  |  |  |  |
|  |  |  |  |  | TH | 59 (21) | |  |  |  |  |  |  |
|  |  |  |  | ddI/d4T/EFV/NFV | Physical Health (PHS) | 48 (12) | | 1, 50.0 | -0.17 | No | No | |  |
|  |  |  |  |  |  |  |  | 2, 48.1 | -0.01 | No | No | |  |
|  |  |  |  |  |  |  |  | 3, 50.0 | -0.17 | No | No | |  |
|  |  |  |  |  | Mental Health (MHS) | 50 (9) | | 1, 50.0 | 0.00 | No | No | |  |
|  |  |  |  |  |  |  |  | 2, 49.5 | 0.06 | No | No | |  |
|  |  |  |  |  |  |  |  | 3, 53.4 | -0.38 | No | No | |  |
|  |  |  |  |  | GPH | 42 (26) | | Not reported |  |  |  | |  |
|  |  |  |  |  | Pain | 74 (29) | |  |  |  |  |  |  |
|  |  |  |  |  | PF | 79 (26) | |  |  |  |  |  |  |
|  |  |  |  |  | RF | 70 (43) | |  |  |  |  |  |  |
|  |  |  |  |  | SF | 73 (32) | |  |  |  |  | |  |
|  |  |  |  |  | MH | 61 (20) | |  |  |  |  | |  |
|  |  |  |  |  | EF | 58 (26) | |  |  |  |  | |  |
|  |  |  |  |  | HD | 71 (28) | |  |  |  |  | |  |
|  |  |  |  |  | CF | 80 (23) | |  |  |  |  | |  |
|  |  |  |  |  | QOL | 59 (24) | |  |  |  |  | |  |
|  |  |  |  |  | TH | 59 (21) | |  |  |  |  | |  |
| Huang et al. (2008) | • Prospective RCT (secondary analysis of RESIST-1 and RESIST-2 data)  • To assess the treatment effects of TPV/r versus CPI/r on HRQL and the relationship between HRQL and AEs observed across both treatment arms | • N=984 (511 in TPV/r arm, 473 in CPI/r arm)  • HIV-1-infected adults with 3 months or longer previous triple ART experience, 2 or more previous PI regimens, HIV-1 RNA 1000 copies per mL or greater, and genotypically demonstrated primary resistance to PIs | • Baseline • 48 weeks • Other Instruments: None | TPV/r (tipranavir / ritanovir) | PHS | 48.0 (11.3) | | 48.2 | -0.02 | Not measured | No | | • MOS-HIV scale and summary scores were significantly lower (in the range of -0.5 to -4.4 points for mild, -1.8 to -6.3 points for moderate, and -0.3 to -13.4 points for severe AEs) compared with scores observed in patients experiencing no AEs (*P*<0.05).  • Increased AE severity resulted in larger reductions in HRQL across all subscale and summary scores with the exception of the CF and MHS scores.  • AEs were more strongly associated with physical health aspects of HRQL (range of scores across AE severity levels for RF -2.5 to -13.4 points; SF -2.9 to -11.4 points; Pain -4.4 to -8.2 points and PF -1.9 to -8.9 points) compared with mental health aspects (only overall QOL saw a major change in scores across AE severity levels -3.0 to -7.8 points), regardless of severity of AE.  • HRQL reductions were more pronounced for the PHS scores (range of scores across AE severity levels -1.5 to -4.3 points) than for the MHS scores (range of scores across AE severity levels -0.8 to -2.0 points). |
|  |  |  |  |  | MHS | 47.8 (10.5) | | 49.2 | -0.13 |  | No | |  |
|  |  |  |  |  | GPH | 48.0 (11.3) | | 48.7 | -0.06 |  | No | |  |
|  |  |  |  |  | Pain | 72.7 (25.9) | | 75.0 | -0.09 |  | Yes | |  |
|  |  |  |  |  | PF | 74.9 (25.6) | | 76.3 | -0.05 |  | No | |  |
|  |  |  |  |  | RF | 64.3 (43.3) | | 64.6 | -0.01 |  | No | |  |
|  |  |  |  |  | SF | 78.7 (26.8) | | 79.3 | -0.02 |  | No | |  |
|  |  |  |  |  | MH | 68.4 (19.4) | | 70.2 | -0.09 |  | No | |  |
|  |  |  |  |  | EF | 57.0 (22.2) | | 58.3 | -0.06 |  | No | |  |
|  |  |  |  |  | HD | 71.5 (25.2) | | 78.1 | -0.26 |  | No | |  |
|  |  |  |  |  | CF | 78.3 (21.6) | | 79.9 | -0.07 |  | No | |  |
|  |  |  |  |  | QOL | 65.0 (21.4) | | 66.4 | -0.07 |  | No | |  |
|  |  |  |  | CPI/r (boosted comparitor PI) | PHS | 47.1 (10.9) | | 46.8 | 0.03 | Not measured | No | |  |
|  |  |  |  |  | MHS | 46.9 (10.3) | | 48.6 | -0.17 |  | No | |  |
|  |  |  |  |  | GPH | 47.1 (10.9) | | 47.6 | -0.05 |  | No | |  |
|  |  |  |  |  | Pain | 73.2 (25.2) | | 70.7 | 0.10 |  | Yes | |  |
|  |  |  |  |  | PF | 74.0 (25.2) | | 76.3 | -0.09 |  | No | |  |
|  |  |  |  |  | RF | 59.9 (44.4) | | 62.5 | -0.06 |  | No | |  |
|  |  |  |  |  | SF | 77.5 (26.9) | | 76.3 | 0.04 |  | No | |  |
|  |  |  |  |  | MH | 65.7 (19.7) | | 69.4 | -0.19 |  | No | |  |
|  |  |  |  |  | EF | 54.0 (22.5) | | 57.7 | -0.16 |  | No | |  |
|  |  |  |  |  | HD | 71.5 (25.2) | | 76.8 | -0.21 |  | No | |  |
|  |  |  |  |  | CF | 78.6 (21.9) | | 79.8 | -0.05 |  | No | |  |
|  |  |  |  |  | QOL | 63.6 (21.1) | | 64.6 | -0.05 |  | No | |  |
|  |  |  |  |  |  |  | |  |  |  |  | |  |
| Lafaurie et al. (2008) | • Prospective RCT (secondary analysis of ALIZE data)  • To assess if patients who have tolerated long-term therapy with zidovudine-containing regimens will benefit from a switch to non-zidovudine-containing cART. | • N=158 (81 in maintenance arm, 77 in switch arm)  • HIV-infected adults receiving unchanged cART for at least 6 months consisting of at least one PI plus two NRTIs and who had never received NNRTIs | • Baseline • 48 weeks • Other Instruments: None | Maintenance of a stable PI-containing regimen | PHS | 56.5 (50.0-61.8)* | | -1.04† | 0.24 | Not Measured | No | | • The mean change from baseline of the PCS and MCS at week 48 were -1.04 and +0.0 U in the maintenance arm compared with -1.76 and +1.01 in the switch arm, respectively (P=0.57 and 0.42).  • Specific items such as PF, SF, and EF remained unchanged in both arms of the study during follow-up (data not shown). |
|  |  |  |  |  | MHS | 40.2 (33.8-45.3)* | | 0.00† | 0.00 |  | No | |  |
|  |  |  |  | Switch to a once-daily regimen of EFV/ddI/FTC | PHS | 57.4 (51.5-60.4)* | | -1.76† | 0.53 | Not Measured | No | |  |
|  |  |  |  |  | MHS | 38.3 (33.4-43.6)* | | 1.01† | -0.27 |  | No | |  |
| Sprinz et al. (2006) | • Prospective RCT (PLATO)  • To investigate the effect of substituting LPV/r for the PI or NNRTI component of a HAART regimen in patients experiencing treatment-related Grade-2 side effects | • N=791 (640 in immediate substitution arm, 151 in deferred substitution arm) • HIV-positive adults experiencing Grade-2 side effects of the PI or NNRTI component of their HAART regimen | • Baseline  • 4 weeks • 8 weeks | IS: Immediate substitution with lopinavir/ritonavir 400/200 mg BID | PHS | 49.44 | | 4, 52.06 | -0.37 | Yes | Yes | | • At week 4, patients in the IS group had improvement of 2.6 points in the PHS score (from a baseline of 49.4; *P<* .001) and a 4.1 point improvement in the MHS score (from a baseline of 46.7; *P<*.001).  • At week 4, the effect size for the between-group difference in PHS and MHS scores were 0.45 and 0.56, respectively, suggesting moderate clinically meaningful improvement in the IS group compared to the DS group. The difference between the groups was statistically significant (*P<* .001 for both PHS and MHS scores).  • At week 8, the IS group maintained its improvement in both PHS and MHS scores. The DS group showed some improvement in the MHS score (*P=* .006), but no significant change in the PHS score (*P=*.151).  • Significant between-group differences with respect to the mean change from baseline to week 8 were observed with respect to both PHS score (*P=*.005) and MHS score (*P<*0 001). In addition, significant between-group differences were observed when comparing measurements obtained during the first 4 weeks on LPV/r with respect to PHS (*P=*.011) and MHS (*P=* .003) scores. |
|  |  |  |  |  |  |  |  | 8, 52.38 | -0.37 | Yes | Yes | |  |
|  |  |  |  |  | MHS | 46.70 | | 4, 50.79 | -0.52 | Yes | Yes | |  |
|  |  |  |  |  |  |  |  | 8, 51.34 | -0.56 | Yes | Yes | |  |
|  |  |  |  |  | GPH | 53.06 | | 4, 60.03 | -0.35 |  | Yes | |  |
|  |  |  |  |  |  |  |  | 8, 60.5 | -0.35 |  | Yes | |  |
|  |  |  |  |  | Pain | 72.86 | | 4, 79.46 | -0.30 |  | Yes | |  |
|  |  |  |  |  |  |  |  | 8, 80.43 | -0.31 |  | Yes | |  |
|  |  |  |  |  | PF | 77.53 | | 4, 81.54 | -0.23 |  | No | |  |
|  |  |  |  |  |  |  |  | 8, 82.36 | -0.24 |  | No | |  |
|  |  |  |  |  | RF | 71.96 | | 4, 77.66 | -0.20 |  | Yes | |  |
|  |  |  |  |  |  |  |  | 8, 78.65 | -0.22 |  | Yes | |  |
|  |  |  |  |  | SF | 80.03 | | 4, 85.80 | -0.24 |  | Yes | |  |
|  |  |  |  |  |  |  |  | 8, 86.00 | -0.24 |  | No | |  |
|  |  |  |  |  | MH | 64.55 | | 4, 70.97 | -0.38 |  | Yes | |  |
|  |  |  |  |  |  |  |  | 8, 71.88 | -0.42 |  | Yes | |  |
|  |  |  |  |  | EF | 56.06 | | 4, 64.77 | -0.46 |  | Yes | |  |
|  |  |  |  |  |  |  |  | 8, 65.42 | -0.48 |  | Yes | |  |
|  |  |  |  |  | HD | 71.75 | | 4, 79.26 | -0.38 |  | Yes | |  |
|  |  |  |  |  |  |  |  | 8, 80.92 | -0.46 |  | No | |  |
|  |  |  |  |  | CF | 75.33 | | 4, 79.56 | -0.24 |  | Yes | |  |
|  |  |  |  |  |  |  |  | 8, 81.09 | -0.32 |  | Yes | |  |
|  |  |  |  |  | QOL | 61.84 | | 4, 69.95 | -0.41 |  | Yes | |  |
|  |  |  |  |  |  |  |  | 8, 69.72 | -0.36 |  | Yes | |  |
|  |  |  |  |  | TH | 59.18 | | 4, 71.86 | -0.45 |  | Yes | |  |
|  |  |  |  |  |  |  |  | 8, 71.37 | -0.41 |  | Yes | |  |
|  |  |  |  | DS: Deferred (week 4) substitution with lopinavir/ritonavir 400/100 mg BID | PHS | 50.32 | | 4, 49.71 | 0.09 | No | Yes | |  |
|  |  |  |  |  |  |  |  | 8, 51.12 | -0.10 | No | Yes | |  |
|  |  |  |  |  |  |  |  | 4*, 51.20 | -0.12 |  | Yes | |  |
|  |  |  |  |  | MHS | 48.25 | | 4, 48.03 | 0.03 | No | Yes | | • Significant between-group differences (*P* ≤ .008) favoring the IS group over the DS group were observed at week 8 with respect to the following MOS-HIV domains: GHP (*P<*.001), RF (*P=* .007), EF (*P=*.001), QOL (*P<* .001), and TH (*P=* .008).  • GHP (*P=*.001), Pain (*P=* .028), EF (*P=* .001), and QOL (*P=* .003) were the only individual MOS-HIV domains for which a significant between-group difference was detected when evaluating the first 4 weeks of LPV/r-based therapy for the IS and DS groups. |
|  |  |  |  |  |  |  |  | 8, 50.02 | -0.21 | Yes | Yes | |  |
|  |  |  |  |  |  |  |  | 4*, 50.12 | -0.24 |  | Yes | |  |
|  |  |  |  |  | GPH | 57.64 | | 4, 55.56 | 0.10 |  | Yes | |  |
|  |  |  |  |  |  |  |  | 8, 56.57 | 0.05 |  | Yes | |  |
|  |  |  |  |  |  |  |  | 4*, 58.67 | -0.05 |  | Yes | |  |
|  |  |  |  |  | Pain | 73.60 | | 4, 73.15 | 0.02 |  | Yes | |  |
|  |  |  |  |  |  |  |  | 8, 75.75 | -0.09 |  | Yes | |  |
|  |  |  |  |  |  |  |  | 4*, 75.65 | -0.09 |  | Yes | |  |
|  |  |  |  |  | PF | 76.83 | | 4, 77.98 | -0.07 |  | No | |  |
|  |  |  |  |  |  |  |  | 8, 81.66 | -0.24 |  | No | |  |
|  |  |  |  |  |  |  |  | 4*, 78.67 | -0.10 |  | No | |  |
|  |  |  |  |  | RF | 76.6 | | 4, 71.43 | 0.18 |  | Yes | |  |
|  |  |  |  |  |  |  |  | 8, 75.58 | 0.03 |  | Yes | |  |
|  |  |  |  |  |  |  |  | 4*, 80.15 | -0.12 |  | No | |  |
|  |  |  |  |  | SF | 80.13 | | 4, 80.54 | -0.02 |  | Yes | |  |
|  |  |  |  |  |  |  |  | 8, 83.94 | -0.15 |  | No | |  |
|  |  |  |  |  |  |  |  | 4*, 82.75 | -0.11 |  | No | |  |
|  |  |  |  |  | MH | 66.55 | | 4, 67.35 | -0.05 |  | Yes | |  |
|  |  |  |  |  |  |  |  | 8, 70.7 | -0.24 |  | Yes | |  |
|  |  |  |  |  |  |  |  | 4*, 70 | -0.20 |  | No | |  |
|  |  |  |  |  | EF | 59.49 | | 4, 59.56 | 0.00 |  | Yes | |  |
|  |  |  |  |  |  |  |  | 8, 63.05 | -0.18 |  | Yes | |  |
|  |  |  |  |  |  |  |  | 4*, 62.57 | -0.17 |  | Yes | |  |
|  |  |  |  |  | HD | 73.73 | | 4, 75.36 | -0.08 |  | Yes | |  |
|  |  |  |  |  |  |  |  | 8, 80.02 | -0.32 |  | No | |  |
|  |  |  |  |  |  |  |  | 4*, 78.32 | -0.23 |  | No | |  |
|  |  |  |  |  | CF | 76.83 | | 4, 75.41 | 0.08 |  | Yes | |  |
|  |  |  |  |  |  |  |  | 8, 79.18 | -0.13 |  | Yes | |  |
|  |  |  |  |  |  |  |  | 4*, 80.63 | -0.22 |  | No | |  |
|  |  |  |  |  | QOL | 64.81 | | 4, 62.96 | 0.09 |  | Yes | |  |
|  |  |  |  |  |  |  |  | 8, 65.48 | -0.03 |  | Yes | |  |
|  |  |  |  |  |  |  |  | 4*, 67.38 | -0.13 |  | Yes | |  |
|  |  |  |  |  | TH | 62.58 | | 4, 56.20 | 0.22 |  | Yes | |  |
|  |  |  |  |  |  |  |  | 8, 67.58 | -0.17 |  | Yes | |  |
|  |  |  |  |  |  |  |  | 4*, 74.05 | -0.39 |  | No | |  |
|  |  |  |  |  |  |  | |  |  |  |  | |  |
| Nuesch et al. (2009) | • Prospective RCT (secondary analysis of STACCATO data)  • To investigate anxiety, stress, depression, and quality of life (QoL) within STACCATO, a randomised trial of two treatment strategies: CD4 guided scheduled treatment interruption (STI) compared to continuous treatment (CT) | • N=241 (82 in CT arm, 159 in STI arm) • Adults with chronic HIV-1 infection who were naïve or antiretroviral-experienced with no evidence of pre-existing drug resistance | • Baseline • 24 weeks • 48 weeks • Last time-point visit before restarting cART (LTP) • Other Instruments: DASS (Depression Anxiety Stress Scale) HADS | Continuous treatment (CT) | Physical Health (PHS) | 53.8 (6.6) | | 24, 52.7 | 0.17 | No | No | | • At baseline, the mean MHS score was significantly lower in the STI arm compared to CT arm; the PHS score was similar between arms. • There was a significant improvement of the MHS across time (*P*=0.001), but no difference between arms (*P*=0.17); the PHS did not change during the trial over time (*P*=0.15) nor between arm (*P*=0.45). |
|  |  |  |  |  |  |  |  | 48, 52.5 | 0.20 |  | No | |  |
|  |  |  |  |  |  |  |  | LTP, 52.4 | 0.21 |  | No | |  |
|  |  |  |  |  | Mental Health (MHS) | 51.7 (7.6) | | 24, 52.7 | 0.29 | Yes | Yes | |  |
|  |  |  |  |  |  |  |  | 48, 52.5 | 0.03 |  | Yes | |  |
|  |  |  |  |  |  |  |  | LTP, 52.4 | 0.00 |  | No | |  |
|  |  |  |  | STI (CD4-guided scheduled treatment interruptions--CD4 threshold of 350 cells/µL for interruption/re-initiation of ARV therapy) | Physical Health (PHS) | 54.4 (6.3) | | 24, 53.2 | 0.19 | No | No | |  |
|  |  |  |  |  |  |  |  | 48, 53.2 | 0.19 |  | No | |  |
|  |  |  |  |  |  |  |  | LTP, 53.1 | 0.21 |  | No | |  |
|  |  |  |  |  | Mental Health (MHS) | 49.1 (8.3) | | 24, 48.1 | 0.12 | Yes | Yes | |  |
|  |  |  |  |  |  |  |  | 48, 49.5 | -0.15 |  | Yes | |  |
|  |  |  |  |  |  |  |  | LTP, 50.1 | -0.12 |  | No | |  |
| Powers et al. (2006) | • Prospective RCT (secondary analysis of MTSI data)  • To examine the effect of repeated, long-cycle structured intermittent versus continuous HAART on health-related quality of life (HRQL) and symptom distress in patients with chronic HIV infection and plasma HIV RNA of less than 50 copies/ml. | • N=46 (23 in continuous treatment arm, 23 in intermittent treatment arm) • HIV-positive adults receiving at least a 3-drug HAART regimen | • Baseline  • 4 weeks • 12 weeks • 40 weeks • Other Instruments: Symptom Distress Scale | Intermittent Group (7 cycles of 4 weeks off/8 weeks on HAART) | Physical Health (PHS) | 54.2 (8.3) | | 4, 54.2 | 0.00 | No | Yes | | • The intermittent group had higher mean PHS and MHS scores than the continuous group at baseline and each observation thereafter.  • Mean MOS-HIV subscale scores across time-points for both groups were lowest in the domains of health perceptions (range, 62–68) and energy/fatigue (range, 56–73), with lower mean energy/fatigue scores for the continuous versus intermittent group at each time-point.  • The highest mean subscale scores were in the domains of physical (range, 84–90), social (range, 84–93) and cognitive function (range, 82–86) across time-points. |
|  |  |  |  |  |  |  |  | 12, 55.55 | -0.16 | No | Yes | |  |
|  |  |  |  |  |  |  |  | 40, 54.76 | -0.07 | No | Yes | |  |
|  |  |  |  |  | Mental Health (MHS) | 54.0 (8.8) | | 4, 53.56 | 0.05 | No | Yes | |  |
|  |  |  |  |  |  |  |  | 12, 57.45 | -0.39 | Yes | Yes | |  |
|  |  |  |  |  |  |  |  | 40, 57.22 | -0.37 | Yes | Yes | |  |
|  |  |  |  | Continuous Group (continue regimen for 22 weeks) | Physical Health (PHS) | 53.0 (11.5) | | 4, 53.27 | -0.02 | No | Yes | |  |
|  |  |  |  |  |  |  |  | 12, 52.97 | 0.00 | No | Yes | |  |
|  |  |  |  |  |  |  |  | 40, 54.0 | -0.09 | No | Yes | |  |
|  |  |  |  |  | Mental Health (MHS) | 53.8 (15.7) | | 4, 53.36 | 0.00 | No | Yes | |  |
|  |  |  |  |  |  |  |  | 12, 57.25 | -0.02 | No | Yes | |  |
|  |  |  |  |  |  |  |  | 40, 57.02 | 0.00 | No | Yes | |  |
| Anis et al. (2009) | • Prospective RCT (secondary analysis of OPTIMA data) • To investigate the relative magnitude and duration of impact of AIDS-defining events (ADEs) and non-AIDS serious adverse events (SAEs) on health-related quality of life (HRQL) among patients with advanced HIV/AIDS | • N=368 • Multidrug resistant HIV adults (have failed at least 2 different multidrug regimens, including drugs of each NNRTI, NNRTI, and PI classes, or laboratory evidence of resistance to drugs in each of the 3 classes) | • T1: within 4 wks before event (SAE or ADE) • T2: 0-8 weeks after event • T3: 8-16 wks after event • T4: ≥ 16 wks of event • Other Instruments: HUI3 (Health Utility Index)  EQ-5D | • The trial evaluated the clinical effects of  (1) retreatment intensification to 5 or more anti-HIV drugs, mega-ART, compared with standard-ART consisting of 4 or fewer anti-HIV drugs, and  (2) a 3-month anti-HIV treatment interruption under clinical observation compared with no interruption before treatment reinitiation • Analyses were conducted without regard to treatment allocation in the study | Physical Health Summary (PHS) | 41.56 (11.7) | | SAE T1, 38.14 SAE T2, 36.59 SAE T3, 39.03  SAE T4, 40.01 | Study measured effects between groups, not changes over time | Not Measured | Yes Yes Yes Yes | | • MOS-PHS scores were significantly lower for those that experienced SAE or ADE compared to those who did not at all time points (*P≤*0.01). • MOS-MHS scores were significantly lower among those who experienced SAEs (T1-T3) and ADEs (T2-T4) compared to those who did not (*P≤*0.05). • MOS-PHS and MOS-MHS scores were significantly higher among those with improvement in CD4 compared to those with no improvement (*P≤*0.01). |
|  |  |  |  |  |  |  |  | ADE T1, 36.70 ADE T2, 35.30 ADE T3, 38.06 ADE T4, 38.50 |  |  | Yes Yes Yes Yes | |  |
|  |  |  |  |  |  |  |  | No SAE T1, 41.76 No SAE T2, 42.02 No SAE T3, 41.76 No SAE T4, 42.14 |  |  | Yes Yes Yes Yes | |  |
|  |  |  |  |  |  |  |  | No ADE T1, 41.63 No ADE T2, 41.72 No ADE T3, 41.61 No ADE T4, 41.82 |  |  | Yes Yes Yes Yes | |  |
|  |  |  |  |  |  |  |  | CD4 improv't, 42.19 |  |  | Yes | |  |
|  |  |  |  |  |  |  |  | pVL improv't, 41.52 |  |  | No | |  |
|  |  |  |  |  |  |  |  | No improv't, 41.07 |  |  | Yes | |  |
|  |  |  |  |  | Mental Health Summary (MHS) | 44.68 (11.4) | | SAE T1, 42.71 SAE T2, 42.03 SAE T3, 43.82 SAE T4, 45.48 |  |  | Yes Yes Yes No | |  |
|  |  |  |  |  |  |  |  | ADE T1, 42.91 ADE T2, 40.33 ADE T3, 42.25 ADE T4, 44.28 |  |  | No Yes Yes Yes | |  |
|  |  |  |  |  |  |  | | No SAE T1, 46.20 No SAE T2, 46.37 No SAE T3, 46.18 No SAE T3, 46.19 |  |  | Yes Yes Yes No | |  |
|  |  |  |  |  |  |  | | No ADE T1, 46.05 No ADE T2, 46.15 No ADE T3, 46.07 No ADE T4, 46.16 |  |  | No Yes Yes Yes | |  |
|  |  |  |  |  |  |  | | CD4 improv't, 46.98 |  |  | Yes | |  |
|  |  |  |  |  |  |  | | pVL improv't, 46.03 |  |  | Yes | |  |
|  |  |  |  |  |  |  | | No improv't, 45.22 |  |  | Yes | |  |
| Wu et al. (2006) | • Prospective RCT (secondary analysis of data)  • To evaluate the efficacy of a computerized reminder device, the Disease Management Assistance System (DMAS), in terms of adherence in people with HIV who were initiating or changing a highly active antiretroviral therapy (HAART) regimen | • N=62 (31 in DMAS + education arm, 31 in education only arm) • HIV-positive adults, treatment naïve or changing HAART (with prior history of 3 or less HAART regimens) | • Baseline  • 6 months • Other Instruments: CES-D IADLs (Instrumental Activities of Daily Living) | Disease Management Assistance System (DMAS), a prompting device that verbally reminds patients at medication times and electronically records doses, and a monthly 30 minute adherence educational session | PHS | 45.7 (11.01) | | 44.22 | -0.13 | No^‡^ | Yes | | • The DMAS group scored significantly better at baseline on seven QOL scales that primarily assess mental health (CES-D, IADLs, mental health summary, general health perceptions, QOL, mental health, and health distress).  • There were no differences between groups on the QOL scales at follow-up.  • In an unadjusted analysis, the groups differed statistically significantly on 7 of the 15 QOL scales, primarily those measuring physical function: CES-D, IADLs, physical health summary, general health perceptions, pain, QOL, and role function.  • After adjusting for age, race, gender, baseline CD4 count, and mean adherence, the results were unchanged except that the differences on the QOL scale became only marginally statistically significant (*P*=0.0571).  • The differences between groups generally reflect a combination of improvement in the standard education arm and some deterioration in the DMAS arm over time. |
|  |  |  |  |  | MHS | 49.2 (10.34) | | 49.45 | 0.02 | Yes^‡^ | No | |  |
|  |  |  |  |  | GPH | 51.3 (28.76) | | 46.75 | -0.16 | Yes^‡^ | Yes | |  |
|  |  |  |  |  | Pain | 66.7 (26.29) | | 64.03 | -0.1 | No^‡^ | Yes | |  |
|  |  |  |  |  | PF | 71.0 (24.24) | | 70.67 | -0.01 | No^‡^ | No | |  |
|  |  |  |  |  | RF | 58.1 (43.01) | | 44.10 | -0.33 | No^‡^ | Yes | |  |
|  |  |  |  |  | SF | 72.9 (31.64) | | 75.30 | 0.08 | No^‡^ | No | |  |
|  |  |  |  |  | MH | 71.7 (19.32) | | 75.82 | 0.06 | Yes^‡^ | No | |  |
|  |  |  |  |  | EF | 57.2 (22.38) | | 61.87 | 0.21 | No^‡^ | No | |  |
|  |  |  |  |  | HD | 67.3 (24.66) | | 75.17 | 0.32 | Yes^‡^ | No | |  |
|  |  |  |  |  | CF | 76.0 (23.89) | | 75.20 | -0.03 | No^‡^ | No | |  |
|  |  |  |  |  | QOL | 75.0 (20.41) | | 65.00 | -0.49 | Yes^‡^ | No (*P*=0.05) | |  |
|  |  |  |  | Education (monthly 30 minute adherence educational session) | PHS | 41.2 (12.70) | | 46.99 | 0.46 | No^‡^ | Yes | |  |
|  |  |  |  |  | MHS | 40.7 (12.43) | | 44.89 | 0.34 | Yes^‡^ | No | |  |
|  |  |  |  |  | GPH | 37.1 (25.03) | | 55.14 | 0.72 | Yes^‡^ | Yes | |  |
|  |  |  |  |  | Pain | 56.3 (34.42) | | 66.93 | 0.31 | No^‡^ | Yes | |  |
|  |  |  |  |  | PF | 61.3 (27.35) | | 73.62 | 0.45 | No^‡^ | No | |  |
|  |  |  |  |  | RF | 45.2 (45.38) | | 60.42 | 0.34 | No^‡^ | Yes | |  |
|  |  |  |  |  | SF | 61.9 (34.00) | | 66.25 | 0.13 | No^‡^ | No | |  |
|  |  |  |  |  | MH | 58.2 (23.55) | | 58.90 | 0.03 | Yes^‡^ | No | |  |
|  |  |  |  |  | EF | 51.5 (22.59) | | 58.89 | 0.33 | No^‡^ | No | |  |
|  |  |  |  |  | HD | 47.9 (31.75) | | 61.74 | 0.44 | Yes^‡^ | No | |  |
|  |  |  |  |  | CF | 64.2 (28.70) | | 72.90 | 0.3 | No^‡^ | No | |  |
|  |  |  |  |  | QOL | 56.5 (28.11) | | 64.11 | 0.27 | Yes^‡^ | No (*P*=0.05) | |  |
| **NON-RANDOMIZED CONTROLLED TRIALS** | | | | | | | | | | | | | |
| Shalit et al. (2007) | • Prospective Controlled Trial (QUALITÉ: QUAlity of LIfe and Tolerability after administration of Enfuvirtide-containing HAART)  • To identify baseline patient demographics or disease status variables that may predict QoL improvements in routine clinical settings. Associations between QoL improvements and clinical outcomes such as changes in HIV RNA and CD4 counts were also assessed. | • N=355 (346 included in QOL analysis) • Treatment experienced, enfuvirtide-naïve, HIV-infected adults and adolescents (age ≥16 years) with CD4 counts >50 cells/mm^3^ | • Baseline  • 12 weeks • Other Instruments: None | T-20 + ARTs | PHS | | Not reported | 2.21 |  | Yes | | N/A | • Significant improvements were observed in the LSM change from baseline to week 12 scores among most MOS-HIV domains; SF and RF were the only domains in which significant improvements did not occur.  • In a secondary analysis of clinical outcomes, CD4 count change from baseline to week 12 was significantly correlated with mental health improvement (Pearson correlation coefficient 0.1140; *P=* .0322). No other significant correlations were observed between week 12 quality of life improvement and clinical outcomes. |
|  |  |  |  |  | MHS | |  | 2.91 |  | Yes | |  |  |
|  |  |  |  |  | GPH | |  | 6.18 |  | Yes | |  |  |
|  |  |  |  |  | Pain | |  | 6.87 |  | Yes | |  |  |
|  |  |  |  |  | PF | |  | 4.93 |  | Yes | |  |  |
|  |  |  |  |  | RF | |  | 3.89 |  | No | |  |  |
|  |  |  |  |  | SF | |  | 2.78 |  | No | |  |  |
|  |  |  |  |  | MH | |  | 3.64 |  | Yes | |  |  |
|  |  |  |  |  | EF | |  | 6.00 |  | Yes | |  |  |
|  |  |  |  |  | HD | |  | 8.04 |  | Yes | |  |  |
|  |  |  |  |  | CF | |  | 2.23 |  | Yes | |  |  |
|  |  |  |  |  | QOL | |  | 6.86 |  | Yes | |  |  |
|  |  |  |  |  | TH | |  | 11.40 |  | Yes | |  |  |
| Levine et al. (2008) | • Prospective Controlled Trial (CHAMPS-2)  • To evaluate the efficacy and safety of epoetin alfa administered subcutaneously (SC) at more prolonged intervals (every 2 weeks) in maintaining QOL and Hb in anemic HIV-infected patients. | • N=292 (237 reporting QOL measures)  • HIV-1-infected anemic adults receiving at least 4 weeks ARV therapy | • Baseline  • Maintenance Phase Day 1 (MPD1) • 24 weeks (24) • Other Instruments: LASA (Linear Analog Scale Assessment) | Epoetin alfa + iron supplementation (in addition to current ART regimen) | PHS | | 38.5 (12.0) | MPD1, 43.0 | -0.38 | No | | N/A | • All 10 domains of MOS-HIV (plus the health transition item) improved significantly from baseline to day 1 of the maintenance phase [all *P*<0.001 except RF (p 0.048)].  • The MOS-HIV domain with the greatest mean improvement from baseline to day 1 of the maintenance phase was EF (17.0-point increase); the largest improvement for the LASA was the energy subscale (22.0-point increase) [data not shown].  • Improvements in QOL scores were associated with increases in Hb.  • No significant decreases in QOL during the maintenance phase were observed for the following parameters: MOS-HIV general health perception, MOS-HIV physical function, MOS-HIV energy/fatigue. |
|  |  |  |  |  |  |  |  | 24, 43.5 | -0.42 | Yes | |  |  |
|  |  |  |  |  | MHS | | 42.4 (11.2) | MPD1, 47.8 | -0.48 | No | |  |  |
|  |  |  |  |  |  |  |  | 24, 47.5 | -0.46 | Yes | |  |  |
|  |  |  |  |  | GPH | | 35.8 (24.0) | MPD1, 44.6 | -0.37 | Yes | |  |  |
|  |  |  |  |  |  |  |  | 24, 43.8 | -0.33 | Yes | |  |  |
|  |  |  |  |  | Pain | | 59.3 (27.4) | MPD1, 66.5 | -0.26 | Yes | |  |  |
|  |  |  |  |  |  |  |  | 24, 67.6 | -0.30 | Yes | |  |  |
|  |  |  |  |  | PF | | 54.3 (27.9) | MPD1, 63.6 | -0.33 | Yes | |  |  |
|  |  |  |  |  |  |  |  | 24, 63.1 | -0.32 | Yes | |  |  |
|  |  |  |  |  | RF | | 43.9 (45.3) | MPD1, 48.7 | -0.11 | No | |  |  |
|  |  |  |  |  |  | |  | 24, 50.5 | -0.15 | Yes | |  |  |
|  |  |  |  |  | SF | | 56.4 (33.3) | MPD1, 66.4 | -0.30 | Yes | |  |  |
|  |  |  |  |  |  |  |  | 24, 68.1 | 0.35 | Yes | |  |  |
|  |  |  |  |  | MH | | 60.6 (22.8) | MPD1, 66.8 | -0.27 | Yes | |  |  |
|  |  |  |  |  |  |  |  | 24, 67.9 | 0.32 | Yes | |  |  |
|  |  |  |  |  | EF | | 41.9 (24.6) | MPD1, 58.9 | -0.69 | Yes | |  |  |
|  |  |  |  |  |  |  |  | 24, 58.1 | 0.66 | Yes | |  |  |
|  |  |  |  |  | HD | | 56.6 (29.7) | MPD1, 67.0 | -0.35 | Yes | |  |  |
|  |  |  |  |  |  |  |  | 24, 67.1 | -0.35 | Yes | |  |  |
|  |  |  |  |  | CF | | 65.8 (27.8) | MPD1, 72.9 | -0.26 | Yes | |  |  |
|  |  |  |  |  |  |  |  | 24, 73.9 | -0.29 | Yes | |  |  |
|  |  |  |  |  | QOL | | 56.4 (24.2) | MPD1, 66.2 | -0.40 | Yes | |  |  |
|  |  |  |  |  |  |  |  | 24, 64.3 | -0.33 | Yes | |  |  |
|  |  |  |  |  | TH | | 57.4 (24.4) | MPD1, 74.7 | -0.71 | Yes | |  |  |
|  |  |  |  |  |  |  |  | 24, 69.0 | -0.48 | Yes | |  |  |
| Stangl et al. (2007) | • Prospective Controlled Trial (secondary analysis of HBAC: Home-Based AIDS Care Project)  • To (1) to explore whether treatment-naıve individuals initiating HAART in rural Uganda experienced improvements in self-reported QOL during the first 12 months of therapy, and (2) to identify the clinical, psychosocial and sociodemographic factors associated with patient QOL in our study population. | • N=947 (831 at follow-up) • Treatment naïve HIV-positive adults | • Baseline  • 3 months • 6 months • 9 months • 12 months • Other Instruments: CES-D | HAART and weekly home visits by study staff that re-supplied HAART and other drugs, conducted a pill count, and assessed participants’ health | Physical Health (PHS) | | 39.2 (9.8) | 3, 50.6 | -1.16 | Yes | | N/A | • At each follow-up visit, PHS and MHS scores were significantly higher than baseline scores (*P*<0.001).  • Mean scores at months 6, 9 and 12 were significantly higher than scores reported at month three (*P*<0.001). • At months 9 (PHS, *P*=0.015; MHS, *P*<0.001) and 12 (*P*<0.001), mean scores were significantly higher than those reported at month six for both scores, but no significant differences were observed between month 9 and month 12 for either the PHS or MHS score. This indicates that increases in QOL occurred through the first nine months of therapy and then levelled off.  • Improvements in QOL closely tracked improvements in CD4 cell count during the first 12 months of HAART. |
|  |  |  |  |  |  |  |  | 6, 53.0 | -1.41 | Yes | |  |  |
|  |  |  |  |  |  |  |  | 9, 53.6 | -1.47 | Yes | |  |  |
|  |  |  |  |  |  |  |  | 12, 54.2 | -1.53 | Yes | |  |  |
|  |  |  |  |  | Mental Health (MHS) | | 40.0 (11.2) | 3, 50.2 | -0.91 | Yes | |  |  |
|  |  |  |  |  |  |  |  | 6, 53.0 | -1.16 | Yes | |  |  |
|  |  |  |  |  |  |  |  | 9, 54.2 | -1.27 | Yes | |  |  |
|  |  |  |  |  |  |  |  | 12, 54.2 | -1.27 | Yes | |  |  |
|  |  |  |  |  | GPH | | 19.9 (17.1) | 58.1 | -2.23 | Yes | |  |  |
|  |  |  |  |  | Pain | | 39.4 (27.5) | 75.4 | -1.31 | Yes | |  |  |
|  |  |  |  |  | PF | | 59.6 (30.7) | 90.3 | -1.00 | Yes | |  |  |
|  |  |  |  |  | RF | | 49.7 (50.0) | 92.8 | -0.86 | Yes | |  |  |
|  |  |  |  |  | SF | | 55.9 (39.2) | 90.4 | -0.88 | Yes | |  |  |
|  |  |  |  |  | MH | | 58.1 (22.0) | 81.6 | -1.07 | Yes | |  |  |
|  |  |  |  |  | EF | | 43.5 (24.9) | 77.0 | -1.35 | Yes | |  |  |
|  |  |  |  |  | HD | | 55.6 (31.3) | 86.8 | -1.00 | Yes | |  |  |
|  |  |  |  |  | CF | | 66.0 (30.2) | 93.8 | -0.92 | Yes | |  |  |
|  |  |  |  |  | TH | | 48.8 (24.5) | 73.2 | -1.00 | Yes | |  |  |
| **Abbreviations:** CF, cognitive function; COH, concern for own health; EF, energy/fatigue; GPH, general perception of health; HD, health distress; MH, mental health; MHS, mental health summary; PF, physical function; PHS, physical health summary; QOL, quality of life; RF, role function; SF, social function; TH, transitory health | | | | | | | | | | | | | |

**Table B. Data extraction tables for** EQ-5D

| **Citation** | **Study design and objective** | **Study population** | **PRO assessment times and other instruments** | **Treatment / dosing regimen** | **Domain** | **Baseline score** mean (SD) | **Follow-up score (**time, mean) | **Effect Size** | | **Significant difference** | | **Clinical / PRO results** |
| --- | --- | --- | --- | --- | --- | --- | --- | --- | --- | --- | --- | --- |
|  |  |  |  |  |  |  |  |  |  | **Over time** | **Between groups** |  |
| **RANDOMIZED CONTROLLED TRIALS** | | | | | | | | | | | | |
| Carr et al. (2001) | • Prospective RCT • To determine if these are reversible with continued HIV suppression following protease inhibitor substitution | • N=80 (31 in continuation arm, 49 in switch arm) • HIV-infected adults receiving a stable PI-containing therapy for at least 8 weeks with no prior abacavir, NNRTI or adefovir therapy | • Baseline • 24 weeks • Other Instruments: None | Continuation arm: continued current ARV therapy | EQ-5D Index (patient assessed) | 85 | 79 | N/A | Not reported | | No (*P*=0.07) | • Quality of life was significantly greater in the switch arm than in the continuation arm at week 24, with a significant relative decline in the continuation arm. |
|  |  |  |  |  | EQ-5D Index (physician assessed) | 89 | 82 | N/A | Not reported | | Yes |  |
|  |  |  |  | Switch arm: switched from prior PI to pen-label abacavir (300 mg twice daily), nevirapine (200 mg daily for 2 weeks, then 200 mg twice daily), adefovir (60 mg daily, with L-carnitine supplementation 500 mg daily) at baseline, and hydroxyurea (500 mg twice daily) from week 4 | EQ-5D Index (patient assessed) | 81 | 89 | N/A | Not reported | | No (*P*=0.07) |  |
|  |  |  |  |  | EQ-5D Index (physician assessed) | 80 | 88 | N/A | Not reported | | Yes |  |
| Anis et al. (2009) | • Prospective RCT (secondary analysis of OPTIMA data) • To investigate the relative magnitude and duration of impact of AIDS-defining events (ADEs) and non-AIDS serious adverse events (SAEs) on health-related quality of life (HRQL) among patients with advanced HIV/AIDS | • N=368 • Multidrug resistant HIV adults (have failed at least 2 different multidrug regimens, including drugs of each NNRTI, NNRTI, and PI classes, or laboratory evidence of resistance to drugs in each of the 3 classes) | • T1: within 4 wks before event (SAE or ADE) • T2: 0-8 weeks after event • T3: 8-16 wks after event • T4: ≥ 16 wks of event • Other Instruments: HUI3 (Health Utility Index)  MOS-HIV | • The trial evaluated the clinical effects of  (1) retreatment intensification to 5 or more anti-HIV drugs, mega-ART, compared with standard-ART consisting of 4 or fewer anti-HIV drugs, and  (2) a 3-month anti-HIV treatment interruption under clinical observation compared with no interruption before treatment reinitiation • Analyses were conducted without regard to treatment allocation in the study | EQ-5D Index | 0.77 (0.19) | SAE T1, 0.69 SAE T2, 0.69 SAE T3, 0.71  SAE T4, 0.73 | Study measured effects between groups, not changes over time | Not Measured | | Yes Yes Yes Yes | • With the exception of ADE T1, EQ-5D scores were significantly lower for those that experienced SAE or ADE compared to those who did not at all time points (*P≤*0.01). • EQ-5D scores were significantly higher among those with improvement in CD4 compared to those with no improvement (*P≤*0.05). |
|  |  |  |  |  |  |  | ADE T1, 0.71 ADE T2, 0.71 ADE T3, 0.71 ADE T4, 0.70 |  |  |  | No Yes Yes Yes |  |
|  |  |  |  |  |  |  | No SAE T1, 0.76 No SAE T2, 0.77 No SAE T3, 0.76 No SAE T4, 0.77 |  |  |  | Yes Yes Yes Yes |  |
|  |  |  |  |  |  |  | No ADE T1, 0.76 No ADE T2, 0.76 No ADE T3, 0.76 No ADE T4, 0.77 |  |  |  | No Yes Yes Yes |  |
|  |  |  |  |  |  |  | CD4 improv't, 0.76 |  |  |  | Yes |  |
|  |  |  |  |  |  |  | pVL improv't, 0.76 |  |  |  | No |  |
|  |  |  |  |  |  |  | No improv't, 0.76 |  |  |  | Yes |  |
|  |  |  |  |  |  |  |  |  |  | |  |  |
| Wu et al. (2002) | • Prospective RCT (secondary analysis of ACTG 204 clinical trial) • To examine the reliability and validity of the EuroQol (EQ-5D) and MOS-HIV and their responsiveness to HIV-related clinical events | • N=990 (32 in AE group, 857 in No AE group; subset of 62 patients experiencing an opportunistic infection also reported) • Treatment experienced HIV-infected adults with no history of CMV end-organ disease | • Baseline  • 4 weeks • 8 weeks • Each 8 weeks thereafter until end of study (EOS) • Other Instruments: EQ-5D VAS MOS-HIV (data not presented) | • This study compared valacyclovir and acyclovir as prophylactic regimens for cytomegalovirus (CMV) • Analyses were conducted without regard to treatment allocation in the study | EQ-5D Index | 0.80 (0.19) | AE 4, 0.72 | -0.42 | Not reported | | No difference between patients reporting and not reporting AEs | • At baseline, no patients had the lowest possible EQ-5D score, while 28.2% scored the highest attainable score of 1.0 and 75% of patients scored between 0.72 and 1. • Effect size was moderate (0.4) for the EQ-5D Index among patients experiencing an adverse event; effect sizes for all other dimensions were “small” and insignificant (0.05-0.20). • In terms of effect size for all PRO instruments, the MOS-HIV pain scale was most responsive to an AE (-0.70); PHS was moderately responsive (-0.5); smaller effect sizes were found for the EQ-VAS, EQ-5D Index, and MOS quality of life, physical and social function scales (-0.3 to -0.4). |
|  |  |  |  |  |  |  | No AE 4, 0.79 | -0.05 |  |  |  |  |
|  |  |  |  |  | EQ-5D Index (N=62) | 0.6 (0.3) | EOS, 0.6 | -0.2 | No | |  |  |
|  |  |  |  |  |  |  |  |  |  |  |  |  |
|  |  |  |  |  | Mobility | 1.2 (0.4) | Not reported | N/A | Not reported | |  |  |
|  |  |  |  |  |  |  |  |  |  |  |  |  |
|  |  |  |  |  | Self-care | 1.0 (0.2) |  |  |  |  |  |  |
|  |  |  |  |  |  |  |  |  |  |  |  |  |
|  |  |  |  |  | Usual activities | 1.4 (0.5) |  |  |  |  |  |  |
|  |  |  |  |  |  |  |  |  |  |  |  |  |
|  |  |  |  |  | Pain/ discomfort | 1.5 (0.5) |  |  |  |  |  |  |
|  |  |  |  |  |  |  |  |  |  |  |  |  |
|  |  |  |  |  | Anxiety/ depression | 1.5 (0.6) |  |  |  |  |  |  |
|  |  |  |  |  |  |  |  |  |  |  |  |  |
| **PROSPECTIVE OBSERVATIONAL STUDIES** | | | | | | | | | | | | |
| Bucciardini et al. (2008) | • Prospective Observational Study  • To provide information on HRQL in ENF-treated subjects in a routine clinical practice | • N=16  • Treatment-experienced, fusion inhibitor-naïve HIV-infected adults | • Baseline  • 3 months • 6 months • Other Instruments: ISSQoL (Istituto Superiore di Sanità Quality of Life) | ENF was given at standard dosage (90 mg SC injection, BID), along with a selected optimized background regimen | Mobility | 1.5 (0.6) | 3, 1.2 | -0.3 | Not reported | | N/A | • On average, the EQ-5D profile of the enrolled sample subjects improved over time.  • Effect size was moderate (0.5) for the Usual Activities domain at 3 and 6 months; effect sizes for all other dimensions were “small” (0.20–0.40) at both 3 and 6 months. |
|  |  |  |  |  |  |  | 6, 1.1 | -0.4 |  |  |  |  |
|  |  |  |  |  | Self-care | 1.2 (0.4) | 3, 1.1 | -0.2 |  |  |  |  |
|  |  |  |  |  |  |  | 6, 1.0 | -0.3 |  |  |  |  |
|  |  |  |  |  | Usual activities | 1.7 (0.6) | 3, 1.3 | -0.5 |  |  |  |  |
|  |  |  |  |  |  |  | 6, 1.3 | -0.5 |  |  |  |  |
|  |  |  |  |  | Pain/ discomfort | 1.7 (0.6) | 3, 1.4 | -0.3 |  |  |  |  |
|  |  |  |  |  |  |  | 6, 1.4 | -0.3 |  |  |  |  |
|  |  |  |  |  | Anxiety/ depression | 1.9 (0.8) | 3, 1.6 | -0.3 |  |  |  |  |
|  |  |  |  |  |  |  | 6, 1.4 | -0.4 |  |  |  |  |
| Bhargava et al. (2010) | • Prospective Observational Study  • To understand the effects of quality of healthcare services and staff on outcomes taking into account the inter-relationships between plasma HIV RNA levels and CD4 cell counts, and effects of higher CD4 cell count on quality of life indicators | • N=102  • HIV-infected adults receiving ART in Free State Province, South Africa | • Baseline  • 6 months • 12 months • 18 months • Other Instruments: EQ-5D VAS | ENF was given at standard dosage (90 mg SC injection, BID), along with a selected optimized background regimen | HRQL | 2.76 (0.38) | 6, 2.78 | -0.05 | No | | N/A | • Mean EQ-5D scores did not change significantly over time. • Percentages of patients scoring the highest possible value (3) on the EQ-5D in the four survey rounds were 50%, 52%, 55%, and 51%. By contrast, percentages of patients reporting the maximum value (100) for the EQ-5D VAS in the four survey rounds were 3%, 10%, 6%, and 9%. |
|  |  |  |  |  |  |  | 12, 2.77 | -0.03 | No | |  |  |
|  |  |  |  |  |  |  | 18, 2.77 | -0.03 | No | |  |  |
